# Corticosteroids on the Management of Coronavirus Disease 2019 (COVID-19): A Systemic Review and Meta-Analysis

**DOI:** 10.18502/ijph.v49i8.3863

**Published:** 2020-08

**Authors:** Mahmoud YOUSEFIFARD, Kosar MOHAMED ALI, Abbas AGHAEI, Alireza ZALI, Arian MADANI NEISHABOORI, Afshin ZARGHI, Saeed SAFARI, Behrooz HASHEMI, Mohammad Mehdi FOROUZANFAR, Mostafa HOSSEINI

**Affiliations:** 1. Physiology Research Center, Iran University of Medical Sciences, Tehran, Iran; 2. College of Medicine, University of Sulaimani, Sulaimani, Iraq; 3. Social Determinants of Health Research Center, Research Institute for Health Development, Kurdistan University of Medical Sciences, Sanandaj, Iran; 4. Functional Neurosurgery Research Center, Shohada Tajrish Neurosurgical Comprehensive Center of Excellence, Shahid Beheshti University of Medical Sciences, Tehran, Iran; 5. Department of Medicinal Chemistry, School of Pharmacy, Shahid Beheshti University of Medical Sciences, Tehran, Iran; 6. Proteomics Research Center, Shahid Beheshti University of Medical Sciences, Tehran, Iran; 7. Emergency Department, Shohadye Tajrish Hospital, Shahid Beheshti University of Medical Sciences, Tehran, Iran; 8. Department of Epidemiology and Biostatistics, School of Public Health, Tehran University of Medical Sciences, Tehran, Iran

**Keywords:** Coronavirus, Coronavirus infections, Glucocorticoids, Methylprednisolone

## Abstract

**Background:**

We aimed to examine the available evidence regarding the efficacy and safety of corticosteroids on the management of coronavirus disease 2019 (COVID-19), severe acute respiratory syndrome (SARS-CoV) and Middle East respiratory syndrome (MERS-CoV).

**Method:**

An extensive search was conducted in Medline, Embase, and Central databases until the end of March 2020, using keywords related to corticosteroids, COVID-19, SARS-CoV and MERS-CoV. The main outcome was considered to be the mortality rate, length of stay, virus clearance time, symptom improvement, and lung function improvement. The findings are presented as odds ratio (OR) with 95% confidence interval (95% CI).

**Results:**

Fifteen paper compromising 5 studies on COVID-19, 8 studies on SARS-CoV and 2 studies on MERS-CoV were included. One study was clinical trial and the rest were cohort. The analyses showed that corticosteroids were not reduce the mortality rate of COVID-19 (OR=1.08; 95% CI: 0.34 to 3.50) and SARS-CoV (OR=0.77; 95% CI: 0.34 to 1.3) patients, while they were associated with higher mortality rate of patients with MERS-CoV (OR = 2.52; 95% CI: 1.41 to 4.50). Moreover, it appears that corticosteroids administration would not be effective in shortening viral clearance time, length of hospitalization, and duration of relief symptoms following viral severe acute respiratory infections.

**Conclusion:**

There is no evidences that corticosteroids are safe and effective on the treatment of severe acute respiratory infection when COVID-19 disease is suspected. Therefore, corticosteroids prescription in COVID-19 patients should be avoided.

## Introduction

Coronavirus disease 2019 (COVID-19) is a global pandemic, starting since the December of 2019 and spreading to all parts of the world, except the Antarctica. The number of affected patients is significantly increasing ever since, and its mortality rate varies in different regions of the world. The mortality rate was 6% compared to active cases, and 21% compared to closed cases (1).

Current treatments for COVID-19 are supportive and symptomatic including the use of antivirals agents, antibiotics, intravenous interferons and gamma globulins, invasive and non-invasive oxygen therapy, and corticosteroids (2, 3). Corticosteroids are widely used as a therapeutic option in COVID-19 and two previous epidemic of coronavirus related severe acute respiratory infection, severe acute respiratory syndrome (SARS-CoV) and Middle East respiratory syndrome (MERS-CoV) (4, 5). Recently, WHO has prohibited corticosteroids administration as a routine treatment for COVID-19 patients (6). However, corticosteroids may decrease mortality rate in COVID-19 patients, casting doubt over WHO recommendation (7).

As a result, a thorough consensus is yet to be achieved in order to provide adequate evidence to determine whether corticosteroids administration is beneficial in the management of COVID-19 patients or not. Hence, we aimed to answer the important question: Do corticosteroids have any beneficial effects in the treatment of severe acute respiratory infection when COVID-19 disease is suspected?

## Methods

### Study design

PICO was determined as follows: problem (P): COVID-19, SARS-CoV, and MERS-CoV patients; intervention (I): corticosteroids therapy; comparison (C): compared with non-corticosteroids treated patients; and outcome (O): mortality, length of stay, virus clearance time, symptom improvement and lung function improvement.

### Eligibility criteria

All clinical trial and observational studies on assessment of corticosteroids therapy in COVID-19, SARS-CoV, and MERS-CoV patients were included. Exclusion criteria were lack of placebo or control group (non-corticosteroids treated patients), non-coronaviruses related disease, non-viral infection, duplicate reports, and review articles.

### Search strategy

An extensive search was conducted on Medline, Embase, and Central databases, from the inception of databases until the end of March 2020. The search was performed using keywords related to corticosteroids, COVID-19, SARS-CoV, and MERS-CoV. A manual search was performed on Google Scholar, Google, and preprinted articles databases.

### Data extraction

Four independent researchers screened the titles and abstracts. Next, potentially relevant studies were identified, and by assessing the full texts, related articles were included. Then, each researcher reviewed and summarized the articles, independently. The data extracted from the articles included first author name, year of publication, country, study type, sample size, name of administered corticosteroid, dosage and route of administration, duration of treatment and outcomes. Any disagreement was resolved by discussing with a third researcher.

### Outcome

The main outcome of the present study was be the mortality rate. Secondary outcomes included length of stay, virus clearance time, symptom improvement, and lung function improvement.

### Risk of bias assessment

The risk of bias assessment in the present study was performed based on two guidelines; the clinical trials were assessed using Cochrane’s risk of bias tools,(8) and for the observational studies, the National Heart Lung and Blood Institute Quality Assessment Tool for Observational Studies was adopted (9). Two researchers reviewed the articles independently, and assigned one of the low risk, high risk, and unclear risk scores for each item of the instructions. Any disagreement was again resolved using a third researcher’s opinion.

### Quality of evidence

The GRADE approach was used to evaluate the quality of evidence and strength of recommendations (10). In this section, two researchers independently assessed the papers, and at the end, a third researcher resolved any disagreements.

### Statistical analysis

All analyses were performed using STATA 14.0 statistical software. The analysis could only be performed on the mortality data. In this section, the analyses were stratified according to the type of the severe acute respiratory infection, meaning the efficacies of corticosteroids treatment on mortalities following COVID-19, SARS-CoV, and MERS-CoV were evaluated separately.

Odds ratio (OR) with 95% confidence interval (95% CI) was included to perform meta-analysis. Some of the included studies reported a hazard ratio, but since the mortality rate was less than 10% in the included studies, the odds ratio and the hazard ratio were very close to each other and were able to be pooled. Heterogeneity between the studies was evaluated using I^2^ test (I^2^ greater than 50% indicated heterogeneity), and since there existed a heterogeneity, random effect model was used for the analyses. Furthermore, Egger’s test was used to evaluate the publication bias. *P* < 0.05 was considered as the significance level in all of the analyses.

## Results

### Study characteristics

**Fig. 1:**
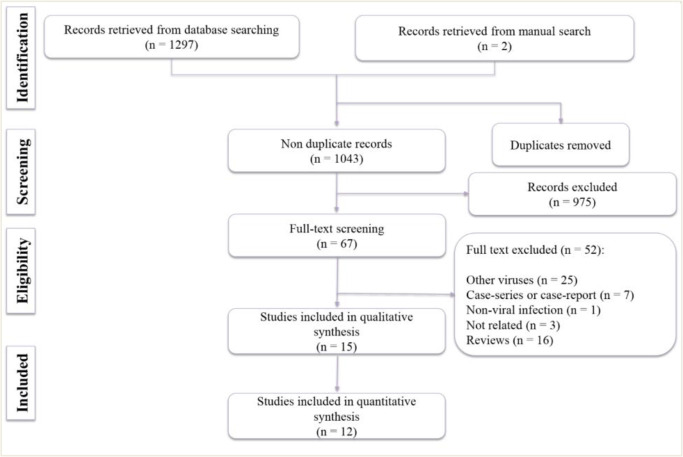
Flow diagram of including of relevant studies

The search in the databases resulted in 1043 non-duplicate articles. After initial screening and reviewing the yielded full texts, 15 studies were included in the present systematic review (Fig. 1) (4, 7, 11–23). The eligible studies include one clinical trial, (15) two prospective cohorts (16, 17) and 12 retrospective cohorts (4, 7, 11–14, 18–23). Five studies were performed on COVID-19 (7, 16, 20, 22, 23) eight studies on SARS-CoV (12–15, 17–19, 21) and two studies on MERS-CoV (4, 11). Except the two studies conducted in Saudi Arabia (regarding MERS-CoV), 13 other studies were performed in China. Eleven articles were in English (4, 7, 11–13, 15, 16, 20–23) whereas four studies were in Chinese (14, 17–19).

These articles contained 4498 patients’ data. All studies were performed on the adult population. The most commonly used corticosteroids were methylprednisolone, prednisolone, hydrocortisone and dexamethasone, respectively. Converted dose into methylprednisolone equivalents ranged from 40 mg to 2000 mg per day. The method of administration was intravenous in 11 studies and not reported in four studies. Furthermore, the treatment time course ranged between one to 21 days; the one-day administration was related to pulse corticosteroids therapy. Mortality was reported in 12 studies, while secondary outcomes including SpO_2_ improvement, need for oxygen therapy, length of hospital stay, virus clearance time, and duration of symptom resolution were reported in eight studies (Table 1).

**Table 1:** Characteristics of included studies

***Author; year; country***	***Country***	***Study type***	***Sample size***	***Corticosteroid group***	***Control group***	***Name of corticosteroid***	***Dose***	***Rout of administration***	***Duration of treatment***	***Outcome***
COVID-19										
Liu; 2020 (16)	China	PCS	78	45	33	NR	40 mg daily	IV	NR	Mortality or disease progression;
Wang; 2020 (20)	China	RCS	46	26	20	MP	1–2 mg/kg	IV	5–7 days	Mortality; SpO_2_ improvement; Need to oxygen therapy
Wu; 2020 (7)	China	RCS	84	50	34	MP	NR	IV	NR	Mortality
Zha; 2020 (22)	China	RCS	31	11	20	MP	40 mg	IV	Median 5 days	Mortality; Symptom duration; Virus clearance time, LOS
Zhou; 2020 (23)	China	RCS	191	57	134	NR	NR	NR	NR	Mortality
SARS-CoV										
Auyeung; 2005 (12)	China	RCS	78	66	12	HC MP Pulse MP	10 mg/kg/day 1–3 mg/kg/day 500–1000 mg/day	IV	NR	Mortality
Chen; 2006 (13)	China	RCS	401	269	132	MP, HC, Dexa	1000–2000 mg/day	IV	NR	Mortality; LOS; Complication
Jia; 2009 (14)	China	RCS	225	134	91	MP, Dexa, P	160–240 mg/day	IV	8–14 days	Symptom improvement, Lung function
Lee; 2004 (15)	China	RCT	16	9	7	HC	300 mg/daily	IV	12 days	Virus clearance time
Meng; 2003 (17)	China	PCS	70	59	11	MP	40 to 640 mg/day	IV	NR	LOS
Song; 2003 (18)	China	RCS	77	60	17	NR	NR	NR	7 days	Mortality
Wang; 2004 (19)	China	RCS	1291	1084	207	MP, Dexa, P	1000–2000 mg	IV	1–14 days	Mortality; LOS
Yam; 2007 (21)	China	RCS	1287	1188	99	HC, MP, P, Pulse MP	NR	IV	15–21	Mortality
MERS-CoV										
Alfaraj; 2019 (11)	Saudi Arabia	RCS	314	NR	NR	NR	NR	NR	NR	Mortality
Arabi; 2018 (4)	Saudi Arabia	RCS	309	151	158	MP, Dexa, P	200 to 300 mg	NR	4 to 14	Mortality; Virus clearance time

COVID-19: Coronavirus disease 2019; Dexa: Dexamethasone; HC: Hydrocortisone; IV: Intravenous; LOS: Length of stay; MERS-CoV: Middle East respiratory syndrome coronavirus MP: Methylprednisolone; NR: Not reported; P: Prednisolone; PCS: Prospective cohort study; RCS: Retrospective cohort study; SARS-CoV: Severe acute respiratory syndrome coronavirus.

### Risk of bias

Risk of bias assessment of the only included clinical trial reveals that allocation concealment, blinding of participants and personnel and selective reporting in the trial have unclear risk of bias. Furthermore, considering the virus clearance time as the main outcome and not assessment of mortality, length of hospital stay or lung function, resulted in the other bias of this study to be considered high risk.

Risk of bias assessments of the observational studies reveals that all of the studies have high risk of bias in sample size justification and blinding of outcome assessor. Besides, only two studies assessed the effects of different doses of corticosteroids in the treatment of SARS-CoV infection (low risk). Other studies are consequently considered as high risk (Table 2).

**Table 2:** Quality assessment of included studies

***Author; Year***	***Item 1***	***Item 2***	***Item 3***	***Item 4***	***Item 5***	***Item 6***	***Item 7***	***Item 8***	***Item 9***	***Item 10***	***Item 11***	***Item 12***	***Item 13***	***Item 14***
**NIH risk of bias tool**														
Alfaraj; 2019	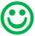	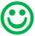	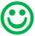	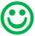	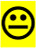	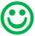	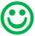	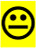	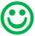	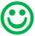	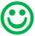	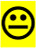	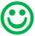	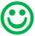
Arabi; 2017	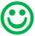	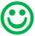	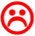	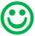	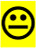	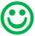	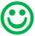	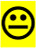	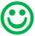	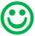	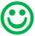	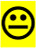	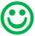	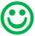
Auyeung; 2005	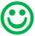	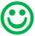	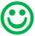	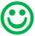	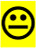	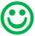	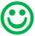	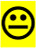	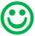	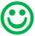	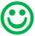	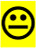	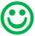	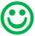
Chen; 2006	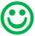	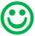	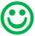	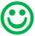	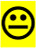	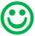	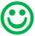	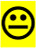	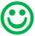	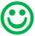	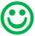	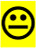	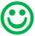	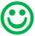
Jia; 2009	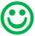	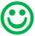	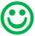	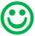	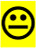	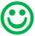	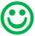	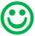	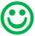	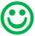	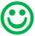	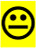	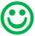	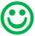
Liu; 2020	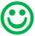	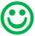	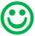	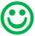	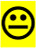	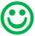	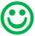	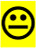	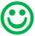	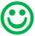	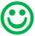	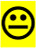	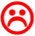	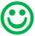
Meng; 2003	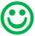	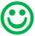	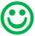	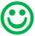	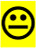	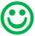	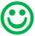	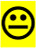	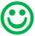	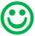	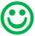	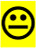	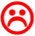	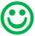
Song; 2003	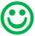	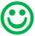	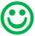	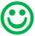	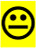	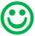	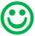	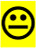	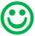	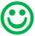	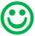	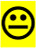	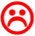	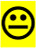
Wang; 2004	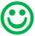	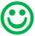	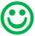	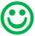	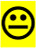	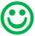	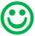	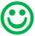	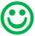	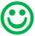	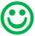	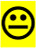	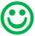	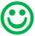
Wu; 2020	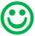	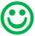	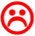	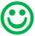	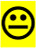	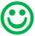	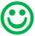	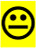	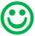	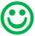	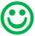	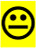	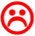	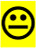
Yam; 2007	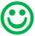	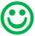	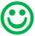	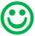	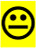	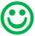	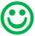	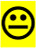	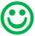	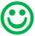	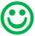	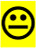	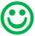	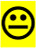
Zha; 2020	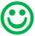	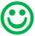	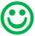	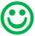	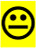	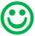	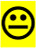	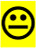	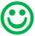	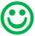	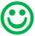	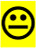	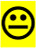	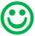
Zhou; 2020	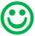	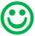	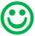	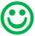	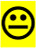	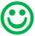	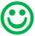	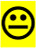	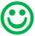	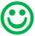	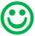	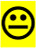	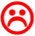	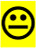
Wang; 2020	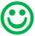	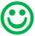	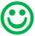	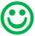	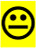	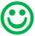	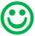	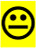	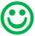	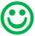	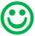	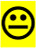	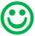	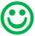
**Cochrane risk of bias tool**														
Lee; 2004	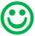	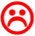	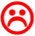	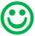	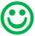	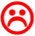	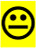	NA	NA	NA	NA	NA	NA	NA

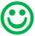
**,** Low risk; 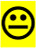
**,** High risk; 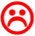
**,** Unclear; NA: Not applicable.

**Items for National Heart Lung and Blood Institute risk of bias tools:** 1. Was the research question or objective in this paper clearly stated?; 2. Was the study population clearly specified and defined?; 3. Was the participation rate of eligible persons at least 50%?; 4. Were all the subjects selected or recruited from the same or similar populations (including the same time period)? Were inclusion and exclusion criteria for being in the study prespecified and applied uniformly to all participants?; 5. Was a sample size justification, power description, or variance and effect estimates provided?; 6. For the analyses in this paper, were the exposure(s) of interest measured prior to the outcome(s) being measured?; 7. Was the timeframe sufficient so that one could reasonably expect to see an association between exposure and outcome if it existed?; 8. For exposures that can vary in amount or level, did the study examine different levels of the exposure as related to the outcome (e.g., categories of exposure, or exposure measured as continuous variable)?; 9. Were the exposure measures (independent variables) clearly defined, valid, reliable, and implemented consistently across all study participants?; 10. Was the exposure(s) assessed more than once over time?; 11. Were the outcome measures (dependent variables) clearly defined, valid, reliable, and implemented consistently across all study participants?; 12. Were the outcome assessors blinded to the exposure status of participants?; 13. Was loss to follow-up after baseline 20% or less?; 14. Were key potential confounding variables measured and adjusted statistically for their impact on the relationship between exposure(s) and outcome(s)?

**Items for Cochrane risk of bias tools:** 1. Random sequence generation. 2. Allocation concealment; 3. Blinding of participant and personnel; 4. Blinding of outcome assessor; 5. Incomplete outcome data; 6. Selective reporting; 7. Other bias

### Quality of Evidence

According the GRADE guideline, the certainly of evidence derived from observational studies is low. We downgraded the level of evidence from low (observational data) to very low due to high risk of indication bias and low sample size of non-corticosteroids group. Our judgment resulted in that sicker patients were more likely to receive corticosteroids than others. In addition, there was a substantial inconsistency among studies and in secondary outcomes. Therefore, overall certainty of evidence in all assessed outcomes is very low (Table 3).

**Table 3:** Quality of evidence based on GRADE guideline

***Outcome***	***Number of studies***	***Design***	***Risk of bias***	***Imprecision***	***Inconsistency***	***Indirectness***	***Publication bias***	***Other consideration***	***Quality of evidences***
Mortality									
COVID-19	5	4 RCS 1 PCS	Serious^1^	Serious^2^	Serious^3^	No serious	No serious	No serious	Very low
SARS-CoV	5	3 RCS 1 PCS 1 RCT	Serious^1^	Serious^2^	Serious^3^	No serious	No serious	No serious	Very low
MERS-CoV	2	2 RCS	Serious^1^	Serious^2^	Serious^3^	No serious	No serious	Serious^4^	Very low
Length of stay									
COVID-19	1	RCS	Serious^1^	Serious^2^	Serious^3^	No serious	Not applicable	Serious^4^	Very low
SARS-CoV	3	2 RCS 1 PCS	Serious^1^	Serious^2^	Serious^3^	No serious	No serious	No serious	Very low
Virus clearance time									
COVID-19	1	RCS	Serious^1^	Serious^2^	Serious^3^	No serious	Not applicable	Serious^4^	Very low
SARS-CoV	1	RCS	Serious^1^	Serious^2^	Serious^3^	No serious	Not applicable	Serious^4^	Very low
MERS-CoV	1	RCS	Serious^1^	Serious^2^	Serious^3^	No serious	Not applicable	Serious^4^	Very low
Symptom and lung function improvement									
COVID-19	2	RCS	Serious^1^	Serious^2^	Serious^3^	No serious	No serious	Serious^4^	Very low
SARS-CoV	2	RCS	Serious^1^	Serious^2^	Serious^3^	No serious	No serious	Serious^4^	Very low

1. Some studies had a high risk of bias

2. Sample size of included studies in non-treated patients was low.

3. There is a considerable heterogeneity.

4. The number of included studies is low

### The effects of corticosteroids administration on the outcomes of respiratory diseases caused by the coronaviruses

#### COVID-19 related mortality

**Fig. 2:**
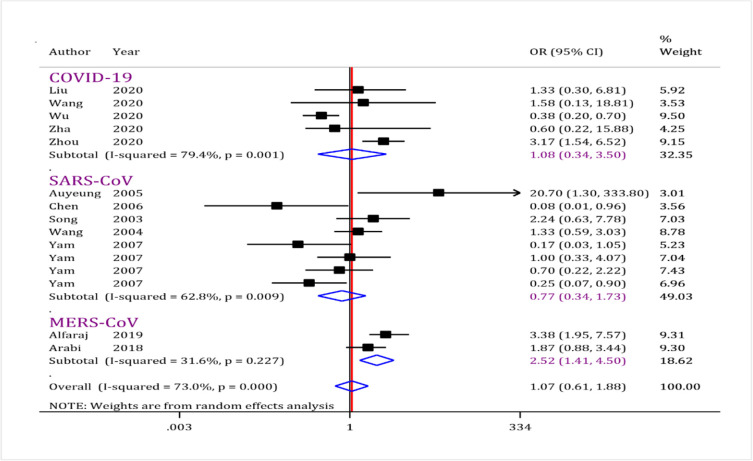
Forest plot for assessment of corticosteroid administration on mortality rate following coronavirus disease (COVID-19), severe acute respiratory syndrome coronavirus (SARS-CoV), and Middle East respiratory syndrome coronavirus (MERS-CoV). The results showed corticosteroids administration did not reduce risk of mortality after COVID-19 and SARS-CoV infection. While, corticosteroids administration increased mortality of MERS-CoV infected patients. CI: Confidence interval; OR: Odds ratio

**Fig. 3:**
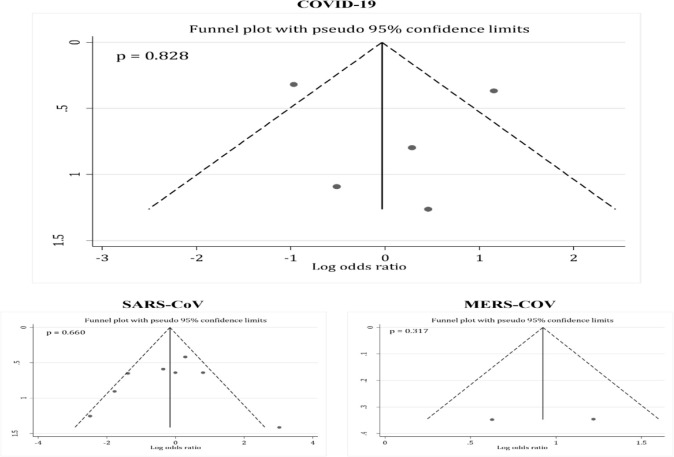
Funnel plot for assessment of publication bias among included studies. There are no evidence of publication bias

Five observational studies (7, 16, 20, 22, 23) had assessed the effects of corticosteroids administration on mortality of the COVID-19 patients (data from 430 patients). The analysis showed that administration of corticosteroids had no beneficial effect in reducing mortality following COVID-19 (OR = 1.08; 95% CI: 0.34 to 3.50; I^2^ = 79.4%; *P*=0.001) (Fig. 2). No publication bias was observed in this section (*P*= 0.828) (Fig. 3).

#### Secondary outcomes in COVID-19

In addition to mortality rate, the efficacy of corticosteroids treatment on SpO_2_ improvement, need for oxygen therapy, duration of symptoms, and length of hospital stay were evaluated in two studies. First, corticosteroids therapy would shorten the need for oxygen therapy and accelerate SpO_2_ improvement (20). However, in another study, virus clearance time (HR = 1.26, 95% CI: 0.58–2.74.55), length of hospital stay (HR = 0.77, 95% CI: 0.33–1.78) and duration of symptoms (HR = 0.86, 95% CI: 0.40–1.83) would not be affected by corticosteroids administration (22).

#### SARS-CoV related mortality

Eight studies (3445 patients) were included in this part of the current meta-analysis (12–15, 17–19, 21). The analyses reveal that corticosteroids administration would not change mortality rate following SARS-CoV infection (OR = 0.77; 95% CI: 0.34 to 1.3; I^2^ = 62.8%; *P*= 0.009) (Fig. 2). No publication bias was observed in this section (*P*= 0.660) (Fig. 3).

#### Secondary outcomes in SARS-CoV

Length of hospital stay was another investigated outcome in the included studies. In the first study, Chen et al evaluated 401 patients and reported that corticosteroids administration would shorten the length of hospital stay (13). However, different doses of corticosteroids did not change the length of hospital stay (17). Finally, Wang et al. observed no relationships between corticosteroids administration and length of hospital stay (19). The disease complications are not related to corticosteroids administration (13). However, corticosteroids alleviated the disease symptoms and improved lung function (14).

In the only included clinical trial, Lee et al performed a double-blinded trial on 16 patients and reported that the virus clearance time, which was directly related to the length of hospital stay, was rather increased when using corticosteroids (15).

#### MERS-CoV mortality rate

Data from two studies (4, 11) were analyzed in this section (623 patients). The analysis revealed that corticosteroids administration increases mortality rates following MERS-CoV infection (OR = 2.52; 95% CI: 1.41 to 4.50; I^2^ = 31.6%; *P* = 0.227) (Fig. 2). No publication bias was observed in this section (*P*= 0.317) (Fig. 3).

#### Secondary outcomes in SARS-CoV

The only evaluated secondary outcome in MERS-CoV section was virus clearance time. Corticosteroids therapy caused a delay in the clearance time in MERS-CoV infection (4).

## Discussion

Current evidence showed that corticosteroids administration do not have beneficial effects in decreasing mortality rate following COVID-19 and SARS-CoV, while these drugs increase the risk of mortality in MERS-CoV patients. In addition, there were significant discrepancy among studies in evaluating the efficacy of corticosteroids in shortening the length of hospital stay, duration of symptom resolution and viral clearance time. Therefore, no evidences exist regarding safety and efficacy of corticosteroids on the treatment of respiratory infection caused by coronaviruses. These results confirmed the WHO conclusion that emphasis corticosteroids should not be used as a routine treatment for COVID-19 patients (6).

Only one double-blinded clinical trial was included in the current review, which did not have the good quality, when its risk of bias was assessed. The study did not provide details of allocation concealment, blinding of participants and personnel. Also, considering virus clearance time as the main outcome and not paying attention to the mortality rate, length of hospital stay, or lung function resulted in the study have a high risk of bias in other bias item. All of the other included studies were cohort. Quality of evidence assessments for these studies showed that serious limitations exist regarding their research methodology. Hence, the findings of the included studies lie within the “very low level of evidence” range.

The use of corticosteroids is not only in doubt in the treatment of respiratory infections caused by coronaviruses, but also there exist numerous meta-analyses, prohibiting its administration in pneumonia caused by influenza. For instance, recently Lansbury et al performed a meta-analysis on 30 studies (1 clinical trials and 29 observational studies) and found that corticosteroids administration increase the risk of mortality in patients with influenza (OR = 3.90; 95%; CI: 2.31–6.60). On the other hand, these drugs result in higher risk for acquiring secondary nosocomial infections (OR = 2.74; 95% CI: 1.51–4.95) (24). In another meta-analysis Ni et al reached a similar conclusion (25). Therefore, it seems that corticosteroids in viral pneumonia is not useful and may worsen the prognosis of the patients.

There still remains unanswered questions, regarding the effects of corticosteroids administration on the outcome of COVID-19. First, is the efficacy of corticosteroids in COVID-19 patients related to the severity of disease? Second, does the efficacy of corticosteroids in COVID-19 patients with acute respiratory distress syndrome (ARDS) differ from non-ARDS patients? For these questions to be responded, we can refer to the studies conducted on influenza. Corticosteroids administration causes an increased risk of mortality and nosocomial infections, both in intensive care unit (ICU) admitted patients and non-ICU patients. In other words, the role of corticosteroids in increasing the risk of mortality is not affected by the severity of disease (26). In addition, a meta-analysis, aiming to evaluate the effects of corticosteroids therapy on outcome of ARDS patients, reported that in subgroups of influenza-related ARDS, corticosteroids increase the risk of mortality (RR = 2.45; 95% CI: 1.40–4.27). More interestingly, administration of corticosteroids for other etiologies of ARDS, such as sepsis-related ARDS and post-operative ARDS, does not affect the mortality rate (27). Also, Zhou et al performed a meta-analysis to assess the effects of corticosteroids administration on the outcomes of influenza-related ARDS, reporting similar findings, and stating that prescription of these drugs not only increases the risk of mortality in influenza-related ARDS, but also increases the risk of acquiring secondary nosocomial infections (28). Although other meta-analyses exist, showing that corticosteroids can decrease mortality rate in ARDS patients (29) but the ARDS population included in these studies were mixed-population (all cause ARDS including trauma, contusion, post-surgery, bacterial and viral), preventing their results to be generalizable to viral respiratory infections.

Finally, only one study was conducted on COVID-19, which performed a bivariate cox regression model, depicting that corticosteroids administration can decrease the mortality rate of ARDS patients following COVID-19. However, the analyses of this study were not adjusted for the potential confounders (7). This is a serious limitation, and has made the results doubtful.

## Conclusion

Corticosteroids administration does not decrease the risk of mortality following COVID-19 and SARS-CoV, while it increases the mortality risk in patients with MERS-CoV. In addition, significant disagreement exists among the studies regarding the efficacy of corticosteroids in shortening the length of hospital stay, duration of symptom resolution and viral clearance time. In general, there is no evidence that corticosteroids are safe and effective in the treatment of respiratory infections caused by coronaviruses. Therefore, corticosteroids prescription in COVID-19 patients should be avoided, unless there are other indication.

## Ethical considerations

Ethical issues (Including plagiarism, informed consent, misconduct, data fabrication and/or falsification, double publication and/or submission, redundancy, etc.) have been completely observed by the authors.
